# Decennial Address

**Published:** 1893-06

**Authors:** Thomas Lothrop

**Affiliations:** Buffalo, N. Y.; Professor of Obstetrics and Vice-President of the Faculty of Medicine


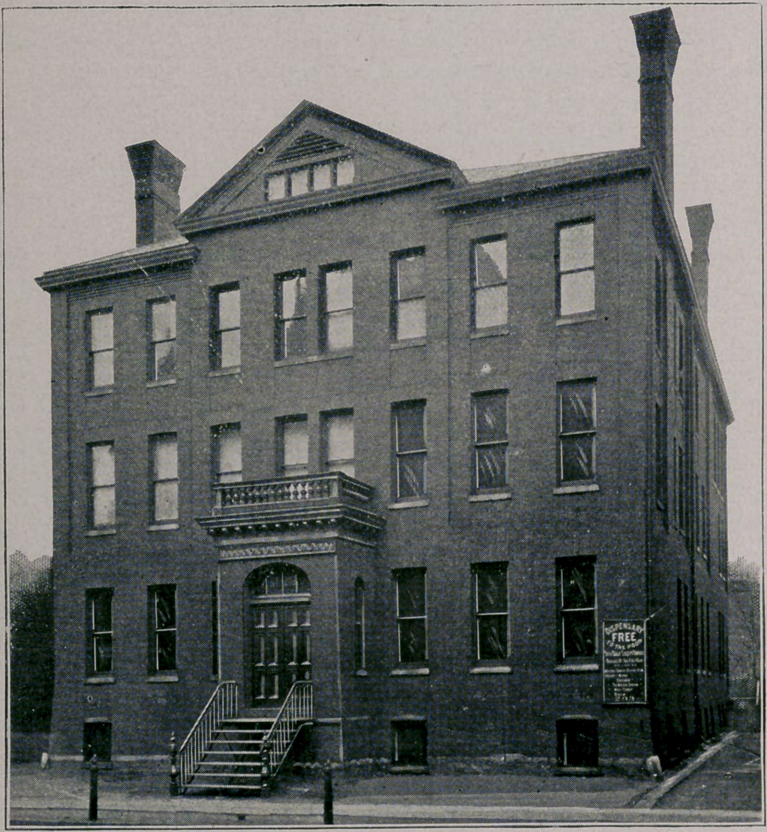

MEDICAL DEPARTMENT—NIAGARA UNIVERSITY.

Buffalo Medical and Surgical Journal

VOL. XXXII. JUNE, 1893. NO. 11.

### DECENNIAL ADDRESS.

DELIVERED AT THE TENTH ANNUAL COMMENCEMENT OF THE MEDICAL DEPARTMENT OF NIAGARA UNIVERSITY, MAY 4, 1893.

BY THOMAS LOTHROP, M. D., Buffalo, N. Y.
Professor of Obstetrics and Vice-President of the Faculty of Medicine.

*Rt. Rev. Chancellor, Mr. President of the University, and Ladies and Gentlemen:*

With the interesting and impressive exercises of this evening we complete the tenth year in the history of this school of medicine. The occasion is one for sincere and hearty congratulation. When, in the course of human events, an epoch is reached which exemplifies the triumph of a principle, in the successful organization of institutions of learning or of beneficence, such occasions may justly be regarded of sufficient importance to call forth congratulatory encomiums for the founders especially, as well as for the community for whose good such beneficent labors were inspired. We regard the decade of years which mark the existence of this school to include one event, at least, in the history of our beautiful city worthy of special note and of generous commendation.

My colleagues have bidden me to give the decennial address at this convocation, in which to portray, briefly, to our assembled guests, the principles which have inspired our labors, and the magnificent results arising therefrom. That this duty should have fallen into other and abler hands would have been the preference of the speaker. To listen, rather than to speak, on an occasion like this, is a modest choice.

The organization and establishment of this school of medicine was the offspring of a necessity. Among the schools of medicine in this State, some of which are sources of pride and admiration to the profession, this school was the last to be legally established. In the medical profession, an agitation has been maintained for many years for higher medical education. This agitation was the natural outcome of abuses within, which finally touched the vital and sacred interests of the public, and then the yet more precious interests of the family and individual. Through a laxity in the laws of the several States, and the indifference in public sentiment, this country has witnessed the establishment of over one hundred medical colleges, which have turned loose thousands of graduates annually, with degrees legalizing them as practitioners, and with both literary and technical training in one of the most humane and comprehensive of the sciences, so defective and inadequate that the medical profession itself, through its representative men, have called aloud for a reform and for advanced standards. Local, State, and National societies have become aroused to take decided action in favor of higher literary or educational training for admission into the profession, of advancement in the curriculum and period of study, for more rigid examinations for the degree of Doctor in Medicine, and for greater inducements for a higher order of ability, as well as of training, to fill the ranks. The reform sought for came from a recognition of defects existing within. The benefits to be derived from this revolution were to be disseminated to the communities and people without. Until the year 1892, the laws of this State required an attendance only on two courses of lectures, of sixteen or more weeks each, with a certificate from some reputable physician certifying to a study of three years, including the lecture term, without any standard of literary qualifications, and with only such a standard for graduation as an interested faculty might impose. The bars of entrance into the field of medicine were let down, and all the rabble rushed in, without regard to fitness, or quality, or aptitude. The demand for three courses of lectures, in three successive years, assumed a practical shape, for the first time in this State, when Niagara University voluntarily and designedly incorporated into its charter, by special legislative enactment, compulsory attendance for the full three years, while the trustees and faculty, actuated by an honest desire for advanced standards, instituted, and for years maintained, a standard of qualifications for admission to its courses far in advance of the regents' academic diploma or medical students' certificate, which became a law in 1891. In justice to an institution in this State—I refer to Syracuse University—let it be said that the three years' course had been, previously to the enactment of this law, maintained, though not absolutely required by its charter. The course, in that college, extends for a period of nine months in each year, and the instruction is mainly recitative, rather than by didactic lectures. The graduates of that school have attained a commendable rank in their examinations for license to practise, before the boards of medical examiners in some of the Western States, noticeably in Illinois, which guards the admission of doctors of medicine into practice with greater restrictions than most of the Western States.

With such objects in its organization, strengthened and protected by the incorporation of this principle in its charter, it would be expected, most naturally, that our efforts, at their inception, would have met with encouragement, here and elsewhere, inasmuch as we aimed to correct abuses, the enormity of which were recognized and publicly acknowledged, and also to elevate the profession above the level of a trade, too often prosecuted on commercial principles. It is with regret and chagrin that we state that an unscrupulous opposition arose, from local and interested sources, which endeavored to stamp out the life of this new-born heir in medical education, to destroy this movement for reform, and to nullify its principles, which subsided only when, by special Act of the Legislature, the organic law of the University was placed beyond the powers of its enemies to reach and destroy.

Every sort and kind of reform, in civil as in professional matters, have to pass through an ordeal of caricature, misrepre sentation and defamation. This has been the experience of the past and will always be so in the future. The same spirit crops out in the history of individuals as well as of nations. It led John Bunyan to the dungeon, Victor Hugo to exile, Paul the Apostle to the Gentiles, to his beheading, and that great central figure in the birth of Christianity, Christ to the cross. Principles in every great revolution of ideas when based on right in the abstract, have outlived its enemies. In this humble movement of ours we do not find an exception to the teachings of history. The trustees and faculty have aimed assiduously and honestly with unselfish motives and self-sacrificing labors, for the one result, the good of the people, whom the medical profession are educated to serve. This day the school stands the monument of honorable effort and labor, and of elevated aims and purposes, and has earned the reputation, at least, of *primus inter pares*. It is but just in this connection to its faculty to state that their labors thus far have received no pecuniary reward, the income having defrayed only the current expenses of the institution, while the modest and convenient building on Ellicott street, erected in 1884, and subsequently enlarged to meet its growing wants, has been paid for from the private purses of its faculty. We have not appealed to the public for subscriptions for the erection of a building of vaunted classical architecture, of commanding proportions and exterior beauty, basing our appeal on past self-sacrificing labors and on a local pride for the development of an institution which has filled the coffers of its teachers while contributing to the reputation of its votaries. With this brief exposition of the principles underlying the organization of this school, the aims of its founders, and the ambitions for its future, it may be proper to state that its success has more than justified our highest anticipations. Commencing with a class of thirteen matriculates in the autumn of 1883, and occupying, through the kindness of the good Sisters of Charity, for its lecture-room the amphitheater of their hospital, the college has advanced annually in attendance on its lectures, in the thoroughness of its didactic and clinical instruction, until, with the session of 1892-93, over sixty students have sought the excellent advantages it offers. This success has not been the outcome of loose methods, of depreciated standards, and underhanded efforts to beguile the unsuspecting and the unwary. The examinations for promotion to the advanced classes have been thorough and impartial, and the exactions for the degree of Doctor in Medicine have been as severe as the faculty and medical examiners could with justice impose.

The trustees, on the recommendation of a separate board of examiners, selected entirely outside of the teaching force, who are the sole judges of the fitness of the applicant for the honors which the University bestows, have conferred the degree of Doctor in Medicine on sixty-three men whose subsequent career has justified the confidence reposed in their ability and fitness for the responsible duties of the profession. Our alumni are not to be solely judged by their quantity. The quality is the surer index of the excellent work we have tried to perform.

Yielding to the demand of the times and actuated by a purpose to keep pace with the rapid strides in scientific research, the pride of the present century, the leading medical schools in this country have extended the period of study to four years, of nine months each. Harvard Medical College, Columbia College, which has the old College of Physicians and Surgeons, of New York, for its medical department; the University of Pennsylvania, the oldest and most reputable of the medical schools of this country; the University of Michigan and Syracuse University, have come up to this standard, and their requirements for admission have also been raised. This school of medicine cannot, with any consistency for its professions, its aims and purposes, long maintain a secondary or subordinate position in a reform so vital to the welfare of the profession and the public. Indeed, while the law of 1891 has compelled all the schools in the State to come up to a three years' course, the position occupied by this school from its very organization, we cannot long maintain, even if we would, an attitude of indifference towards the demands of professional and public sentiment with which the very air is full. We must keep pace with the very best schools in the land, and be in the van-guard of reform, if we are true to the principles proclaimed in our announcement and enunciated in our organic law. Even while placed in the disadvantageous position of requiring a longer period of study than any other school in the State, and, therefore, above and beyond the charge of "catering to an intellectual impatience and a desire for quick and patent results," which a late writer, Mr. Bryce, in his great book on the American Commonwealth, gives as one of the salient intellectual features of our people, the school has acquired a reputation for thorough work, of which all may be proud, while the advantages it offers to the student in medicine in some of its departments are superior to those offered by any other school in the State and in the land. These results have been attained by individual effort, with such aid and encouragement from the parent university and its friends, as they were only too willing to render.

We are anxious to take the advanced step to a four years' course at the earliest possible date. Our city, in its institutions of learning, should not be behind those of neighboring States and Provinces. The Provinces of Canada have set us an example which it would be well to imitate. Not satisfied with the requirements of a four years' course, it is the intention to advance to five years the coming autumn. Their graduates in medicine, some of whom are members of our faculty, show the thoroughness of their training in the advanced position they assume in professional circles in this city. An esteemed medical friend, while visiting recently, Egypt, sent me the prospectus of the medical school at Cairo, under the special guardianship of the government, from which it is learned that a five years' course is required of students in that institution before admitting them to their degree. The comparison of the exactions of a five years' course, demanded of the disciple of Esculapius in Egypt, who is educated to combat the diseases arising from and peculiar to the river Nile, and the standard of three years only, demanded of the novice in medicine who is expected to meet the unsanitary conditions arising from the Hamburg canal, is not flattering to our Christian civilization. It is proper to state in this connection that the State has raised an additional safeguard for the protection of its people by assuming the supervisory work of examining into the fitness of all graduates to practise medicine, through the State Board of Medical Examiners. Our honored townsman, Dr. W. W. Potter, is an influential member of this Board. This movement is in the right direction, and has had, from its inception, the approval of this school. While the University confers the degree of Doctor of Medicine, the State Board is the sole judge of the fitness of the graduate to practise his profession. We think the people have this protection. We hope that a further advance will be made in the right to the educational qualifications required before entering on the study of medicine, by exacting the academic education obtained by graduating at our High Schools as a minimum.

Under the voluntary system peculiar to this country, our institutions of learning are dependent, mainly, upon private beneficence for their endowment. The wealth of our city has not, thus far in its history, been lavished upon its eleemosynary and educational institutions. Let us commend to our rich men the example of Johns Hopkins, of Baltimore, who gave $7,000,000 to found an university and hospital in that city, from which has arisen one of the most complete institutions of learning, and also the most capacious hospital, in this or any other land; of Rockefeller, of Chicago, whose millions have given to that great city an university of almost unlimited resources; of Leland Stanford, Jr., who, in his lifetime, had set apart $20,000,000 of his vast wealth for educational purposes, to found an university which bears his name, which will raise the intellectual standard in that land of gold, and sunshine, and fruit; of the late Ezra Cornell, whose munificent endowment of Cornell University, at Ithaca, has made that magnificent institution an enduring monument to his sagacity and liberality; of Clark, of Worcester, Mass., whose broad-minded character is demonstrated in the establishment of Clark University, in that city, which will add to the intellectual and scientific greatness of

New England, already honored by the vast endowment and grandeur of old Harvard.

Is Buffalo, which, as a center of commerce, has commanded the attention of sagacious business men, especially during the decade of the existence of this school, to remain in the background as an educational center? Are the cereals of the Northwest, the lumber of neighboring States and Provinces, the coal and the minerals of the Keystone State, which are brought to our city as a most convenient distributing point, to be our only pride and boast? We may affirm, with great emphasis, that a city like ours, the eastern terminus of the chain of the great lakes, and the western terminus of one of the most gigantic enterprises ever undertaken by a State, in its system of artificial waterways, should have higher ambitions and aspirations, among its men of wealth, than the mere accumulation of riches, which, in fact and in deed, are only a trust, and not an actual possession. Our sister city, Boston, has been made the center of intellectual culture and power, through its great schools of letters, and science, and art, established by the first settlers of that noble commonwealth, at Cambridge.

The above are a few examples of a wise disposition of wealth for the benefit of succeeding generations. Is it too much to expect that our city, having reached, in population and material wealth, a position of commanding importance, should now turn attention to the educational and moral improvement of its people? Foundations are already laid upon which to erect, through private munificence, a superstructure as grand as the fair reputation of this beautiful city should command. We beg for a kind remembrance for this school of medicine in the hearts of this people. Unselfish labors, from a body of accomplished and educated medical men, have demonstrated that one of the best-equipped schools of medicine can be maintained here. But we need an endowment to enable us to make the advances to which we aspire, and we feel that we have the right, indeed, that it is our bounden duty, to appeal for a recognition and appreciation of our work from those whose accumulations in commerce have been greater than ours in the profession of our choice.

The future of this important work, in a degree at least, rests with the alumni, who, on account of our youth, are but few, though of increasing influence and reputation. We add to this body this evening a class of ten men, the decennial class, who are admitted to their chosen calling, after a course of instruction which we regard to be sufficiently severe and thorough to test their capacity and aptitude for their life-work. We hope we have succeeded in impressing upon their minds a high ideal, towards which to direct their ambitious labors. Our curriculum of study comprehends the fundamental principles of the science of medicine, with such practical work at the bed-side as our ample clinical facilities afford. Herein we deal with the effects of disease, or rather of pathological conditions upon our physical organizations. A thorough knowledge of the structure and functions of the body is necessary before such alterations and changes made by disease can be fully appreciated and understood. But in the physical organization of man, the creative power has superadded a higher principle, the "breath of life," the *vis vitæ* a mysterious entity, as high above human comprehension as is the source from whence it came. The thoughtful physician witnesses the manifestation of this life principle on the dust of which the body is composed. Remedial agencies, wisely applied, modify, relieve, or cure morbid conditions into which our bodies fall. But the influence of this higher principle of life on organic matter is a study of increasing interest as experience accumulates. The good physician, therefore, affiliates naturally with him who ministers in spiritual things, and must recognize the intimate relations existing between the material and the immaterial. Let it be said in justice to the pious men who constitute the governing body of this University, that the only restriction they have imposed upon the instruction imparted to the young men in this school is that this department shall not, through its faculty, be the instrument of disseminating unbelief, agnosticism, or any of the materialistic or rationalistic philosophies which flood and curse our land. Over the portals of the operating-room in one of the large hospitals of Europe a celebrated surgeon has engraved the following inscription: "I dress the wound, God heals it." May I hope that this motto, which embodies an everlasting and eternal principle, may be the inspiration of the decennial class which we send forth to-night.

Young men, we have thus far, since you came under our instruction, stood in the position of your fathers in medicine. Tonight we become your elder brothers. You are born into a new life, than which there is nothing on earth higher, and purer, and nobler. We beg you to prove worthy custodians of these sacred trusts, that your lives may exemplify the highest moral and religious principles, and the world be made the better by this your admission into the noblest profession to which you can consecrate your energies. On behalf of my colleagues, I bid you "God speed," and, in conclusion, will say to each one of you, "Fare thee well."

